# Income Gradient in Renal Disease Mortality in the United States

**DOI:** 10.3389/fmed.2017.00190

**Published:** 2017-11-06

**Authors:** Shervin Assari, Maryam Moghani Lankarani

**Affiliations:** ^1^Center for Research on Ethnicity, Culture, and Health, School of Public Health, University of Michigan, Ann Arbor, MI, United States; ^2^Department of Psychiatry, School of Public Health, University of Michigan, Ann Arbor, MI, United States; ^3^Medicine and Health Promotion Institute, Tehran, Iran

**Keywords:** socioeconomic status, hypertension, diabetes, obesity, deaths, renal diseases

## Abstract

**Background:**

Non-communicable diseases and associated mortality follow a social gradient and chronic kidney disease is not an exception to this rule. Intermediate behavioral and medical factors that may explain such social gradients are, however, still unknown.

**Objectives:**

Using nationally representative data in the United States, this study was conducted to investigate the mediating effect of medical and behavioral risk factors on the association between socioeconomic status (SES) and renal disease mortality.

**Patients and methods:**

Americans’ Changing Lives Study (ACL), 1986–2011, is a 25-year nationally representative prospective cohort study. ACL followed 3,361 adults for up to 25 years. Income, education, and unemployment were the main predictors of interest. Death due to renal disease was the main outcome. Health behaviors (smoking, drinking, and exercise) and medical risk factors (diabetes, hypertension, and obesity) were the mediators. Cox proportional hazards models were used for data analysis.

**Results:**

Higher income (HR = 0.75; 95% CI = 0.62–0.89) was associated with lower risk of death due to renal disease over the 25-year follow-up period. Although health behaviors and medical risk factors at baseline were also predictors of the outcome, they failed to explain the effect of income on death due to renal disease. That is, income was associated with death due to renal disease above and beyond all potential mediators including behavioral and medical risk factors.

**Conclusion:**

Socioeconomic inequalities in the United States cause disparities in renal disease mortality; however, such differences are not due to health behaviors (smoking and drinking) and medical risk factors (hypertension and diabetes). To reduce disparities in renal disease mortality in the United States, policies should go beyond health behaviors and medical risk factors. While programs should help low-income individuals maintain exercise and avoid smoking, reduction of income disparities should be regarded as a strategy for reduction of disparities in renal disease mortality. By increasing minimum pay and minimizing the income gap, we may reduce disparities in renal disease mortality.

## Background

In the United States, socioeconomic status (SES) is a major determinant of mortality due to renal diseases (1–8). While SES indicators such as income, education, employment, wealth, and race determine burden of chronic kidney disease (3–6), it is still unknown whether these disparities are due to health behaviors (e.g., smoking, drinking, and exercise) and medical risk factors (e.g., obesity, diabetes, and hypertension) or not (9–11).

We have previously compared various countries for the effects of SES, health behaviors, and medical risk factors on well-being of populations. Across 15 countries, the United States was the only country in which the effect of income on subjective health is fully explained by medical risk factors. In none of the countries studied, income disparities in subjective health could be fully explained by health behaviors (11). In a recent study, SES (income), health behaviors (smoking, drinking, and exercise), and medical risk factors (diabetes and hypertension) could explain the racial disparities in renal disease mortality in the United States (9). In another study, SES and chronic medical conditions explained racial differences in all-cause mortality in the United States (10). In another study, income was the only SES indicator which universally determined life expectancy across social groups (12). All these studies suggest that income may have a more salient role as a social determinant in the United States compared to other countries.

The effects of SES, health behaviors, and medical risk factors on renal disease mortality are complex (8). Behavioral risk factors may be proximal explanatory factors (i.e., mediators) of SES disparities in renal disease burden. We know that behavioral risk factors such as physical activity, smoking, and drinking—which influence development and progression of renal diseases (13–16)- follow a SES gradient (17–20). Medical risk factors (hypertension, diabetes and obesity) may also explain SES disparities in renal disease. As chronic medical conditions (CMC) such as hypertension and diabetes are leading causes of kidney failure (1), SES disparities in burden of renal disease may be at least in part secondary-to-higher prevalence of CMC among low SES individuals (21, 22). Hypertension (23), diabetes (24), and obesity (25), which are known causes of kidney disease, are all more common in low SES populations (24, 26–28). The role of diabetes (29, 30) and hypertension (31, 32) as etiologic factors in development of chronic kidney disease are well established. Obesity—which is also common among low SES and minorities—may also explain why risk of chronic renal disease is higher among low SES groups (33). Two recent studies have shown that the effect of depression on renal disease mortality was smaller for Whites than Blacks (2). However, medical risk factors (e.g., diabetes, hypertension, obesity) had a stronger long-term effect on renal disease mortality across various SES groups (7).

For at least four reasons, more studies are needed on the complex links between SES, health behaviors, medical risk factors, and disparities in renal disease mortality in the United States. First, there is considerable evidence suggesting that contribution of SES, behaviors, and CMC to health disparities are not universal but vary across countries (11, 34–38). For instance, income may have a stronger role on health disparities in the United States than in other countries (11). Second, only a handful of studies have ever focused on mechanisms that link SES to renal disease mortality (2, 9, 10). Third, not only distribution of the medical risk factors of renal disease depends on SES and race, but also their fatality depends on SES factors (33, 39–42). Last, but not least, most research on this topic has used a local sample, and most prospective studies have used a short-term follow-up period. Thus, there is a need for additional studies that follow a national sample for a long-term period.

## Objectives

To better understand mechanisms behind SES gradient in renal disease mortality in the United States, we tested whether or not behavioral and medical risk factors explain SES inequalities in mortality due to renal diseases or not.

## Methods

### Design and Setting

This is a 25-year longitudinal study. We used data from the Americans’ Changing Lives (ACL), 1986–2011. ACL is a state-of-the-art nationally representative US cohort study conducted by the University of Michigan. Detailed information on the study design is available elsewhere (43, 44).

### Participants and Sampling

The ACL used a stratified multistage probability sample of US adults aged 24 or above. The study recruited 3,617 non-institutionalized respondents representing 70% of sampled households and 68% of sample individuals at baseline. The ACL oversampled those 60 years old and older, and Blacks.

### Measures

Data on demographic, SES, behavioral, and medical characteristics were measured at baseline (year 1986) during a face-to-face interview.

#### Socioeconomic Status

The main predictors were two SES indicators, namely, education and income. Education was operationalized as number of schooling years. Income was operationalized as a 10-level categorical variable (<$5K, $5–9K, $10–14K, $15–19K, $20–24K, $25–29K, 30–39K, $40–59K, $60–79K, and $80K+). Education and income were both treated as continuous measures.

#### Race

In this study, race was defined as non-Hispanic Black or non-Hispanic White based on a coding of self-reported items asking about Hispanic ethnicity, nativity, and racial category.

#### Demographic Factors

Demographic indicators included age (a continuous variable as number of years since birth) and gender (men as the reference category).

#### Medical Risk Factors

Self-reported data were collected on history of hypertension and diabetes at baseline. Using separate items, all participants reported whether in their lifetime a health care provider had ever told them that they had hypertension or diabetes (44, 45).

#### Obesity

Body mass index (BMI) was measured based on self-reports of weights and heights, originally collected in pounds and feet/inches, respectively. We defined obesity as a BMI ≥ 30 kg/m^2^ (46). Although BMI based on self-reported weight and height results in an underestimation of BMI (47, 48), it strongly correlates with BMI based on direct measures (49).

#### Health Behaviors

Self-reported data were collected on smoking (current smoker vs. other), drinking (current drinker vs. other), and exercise (frequency of physical activities) using three single-item measures. Item for smoking read, “Do you smoke cigarettes now?”. Item for drinking read, “Do you ever drink beer, wine, or liquor?”. Item for exercise read, “How often do you engage in active sports or exercise—would you say often, sometimes, rarely or never?”. Response items for the first two items were yes and no, and response scale for the third item was (1) often, (2) sometimes, (3) rarely, and (4) never. Smoking and drinking were treated as dichotomous variables; exercise was treated as a continuous measure.

#### Mortality due to Renal Diseases

The main outcome of interest was time of death from renal diseases. Mortality data from mid-1986 through 2011 were obtained through the National Death Index (NDI), death certificates, or the informants. In majority of our deceased participants, time and cause of death could be verified with a death certificate. In only a handful of cases, death information could not be verified with death certificates. In these cases, we reviewed the information carefully, and actual death was certain in all cases. In only a few cases, the death date was ascertained from the informants or the NDI report in lieu of a death certificate (45, 50). Only in a few cases, cause of death was coded as missing, when death certificate or NDI were not available. Respondents who died due to other causes were censored at the time of death. Time of death was registered as number of months from time of enrollment to the study to time of death, based on the month of death and the month of baseline interview.

ICD-9 and ICD-10 codes (51, 52) were used, depending on which was current at the time death was recorded. For ICD-9 codes, the following codes were considered as renal death: 650 (acute glomeronephritis and nephrotic syndrome), 660 (chronic glomeronephritis, nephritis, and nephropathy, not specified as acute or chronic, and renal sclerosis, unspecified), 670 (renal failure, disorders resulting from impaired renal function, and small kidney of unknown causes), and 680 (infections of kidney). For ICD-10 codes, the following codes were considered: 97 (nephritis, nephrotic syndrome, and nephrosis), 98 (acute/rapidly progressive nephritic and nephrotic syndromes), 99 (chronic glomerulonephritis, nephritis, and nephropathy not specified as acute or chronic, and renal sclerosis unspecified), 100 (renal failure), 101 (other disorders of kidney), 102 (infections of kidney), and 104 (inflammatory diseases of female pelvic organ).

### Ethics

The current study followed the tenets of the Declaration of Helsinki. University of Michigan Institutional Review Board (IRB) approved the study protocol. We received written consent from all participants. Data were kept fully confidential.

### Statistical Analysis

Stata 13.0 (Stata Corporation, College Station, TX, USA) was used to conduct the univariate, bivariate, and multivariable analyses. We accounted the stratification and clustering of the sample. SEs were estimated using Taylor series linearization. We considered *p*-values <.05 as statistically significant. We reported adjusted hazard ratios (HRs), associated SEs, and 95% confidence intervals (CIs).

For multivariable analysis, multiple proportional hazards models were run to the pooled sample. Proportional hazards models require a binary outcome (renal death) and time to the event or rime to censoring [number of months between baseline to occurrence of death, loss to follow-up, or end of follow-up (2011)]. Renal death was coded zero if the respondent did not die, or died from any other causes. SES indicators (income, education, and employment) were the main predictors of interest. Health behaviors (smoking, drinking, and exercise) and medical risk factors (hypertension, diabetes, and obesity) were potential mediators.

## Results

### Descriptive Statistics

Table 1 summarizes the descriptive statistics for the sample. There were more women in the study than men. Mean age of the participants was 48 years. In total, 14% of the participants were obese, 30% of the participants were smokers, and 60% reported drinking. From all participants, 0.5% died due to renal diseases.

**Table 1:** Summary of descriptive statistics in the sample.

	Mean (SE)	95% CI
Age	47.79 (0.53)	46.72–48.86
Education	12.53 (0.10)	12.34–12.73
Income	5.41 (0.09)	5.22–5.60
Exercise	0.02 (0.03)	−0.03–0.07
Chronic medical conditions	5.73 (0.00)	4.80–6.82

	**% (SE)**	**95% CI**

Gender		
Male	47.26 (0.01)	44.86–49.68
Female	52.74 (0.01)	50.32–55.14
Obesity	14.47 (0.01)	12.86–16.24
Smoking	30.45 (0.01)	27.81–33.23
Drinking	60.02 (0.02)	56.68–63.26
Renal disease mortality	0.52 (0.00)	0.26–1.03

### Proportional Hazard Models

Table 2 shows the results of five Cox proportional hazard regression models with renal disease mortality as outcome. As Model 1—a model which only included age, gender, race, and SES—shows, income (HR = 0.75; 95% CI = 0.62–0.89) was associated with risk of death due to renal disease. Models 2–4 show that health behaviors and medical risk factors did not mediate the effect of income on our outcome. However, health behaviors (drinking) and medical risk factors (hypertension and diabetes) did predict the outcome. That is, income was associated with death due to renal disease above and beyond all potential mediators including behavioral and medical risk factors.

**Table 2:** Summary of Cox regressions on socioeconomic factors and deaths due to renal disease in the United States.

	Model 1 (SES)		Model 2 (SES + behavioral risk factors)		Model 3 (SES + medical risk factors)		Model 4 (SES + behavioral and medical risk factors)	
			
	HR (SE)	95% CI	HR (SE)	95% CI	HR (SE)	95% CI	HR (SE)	95% CI
Income	0.75 (0.07)	0.62–0.89	0.79 (0.07)*	0.66–0.96	0.75 (0.08)**	0.61–0.92	0.79 (0.09)*	0.62–0.99
Race (Blacks)	2.38 (1.11)^#^	0.93–6.07	1.99 (1.01)	0.71–5.55	1.52 (0.76)	0.56–4.16	1.81 (0.71)	0.82–4.00
Age	1.08 (0.01)	1.06–1.10	1.08 (0.01)***	1.06–1.10	1.07 (0.02)***	1.03–1.10	1.07 (0.01)***	1.05–1.10
Gender (women)	0.49 (0.34)	0.12–2.00	0.32 (0.27)	0.06–1.81	0.40 (0.29)	0.09–1.72	0.30 (0.21)^#^	0.07–1.26
Education (years)	0.76 (0.40)	0.26–2.21	0.54 (0.38)	0.13–2.23	0.56 (0.32)	0.18–1.77	0.51 (0.24)	0.19–1.33
Smoking	–	–	3.52 (2.26)^#^	0.96–12.86	–	–	3.66 (1.89)*	1.29–10.35
Drinking	–	–	0.10 (0.09)*	0.02–0.60	–	–	0.14 (0.08)**	0.04–0.47
Exercise	–	–	0.67 (0.16)^#^	0.41–1.07	–	–	0.73 (0.18)	0.44–1.21
Obesity	–	–	–	–	2.23 (1.40)	0.63–7.91	1.71 (0.85)	0.63–4.65
Hypertension	–	–	–	–	3.36 (1.63)*	1.27–8.91	2.94 (1.32)*	1.19–7.26
Diabetes	–	–	–	–	7.58 (4.33)***	2.40–23.95	6.55 (2.83)***	2.74–15.64

^#^p < 0.1; *p < 0.05; **p < 0.01; ***p < 0.00.1.

*SES, socioeconomic status*.

## Discussion

According to our findings, low SES (low income) increases risk of death due to renal disease over a 25-year period, and this effect cannot be explained by SES differences in health behaviors (smoking, drinking, and exercise) and medical risk factors (diabetes, hypertension, and obesity) at baseline. Although health behaviors and medical risk factors influence risk of the outcome, they do not explain the income gradient in renal disease mortality.

Similar to their effects on a wide range of health outcomes (53–55), this study provided support for SES (income) as a distal determinant of renal disease mortality in the United States. According to the Link and Phelan’s Fundamental Cause Theory (FCT), SES is a root cause of health and illness (56–58). This theory provides four essential features for the effect of SES as a fundamental cause of health inequalities (59). First, the effect of SES is not limited to specific health problems as it influences most health outcomes. Second, the effect of SES on health is through a wide range of mechanisms. Third, SES as a proxy of access to materialistic and human resources reduces health risks and delays or minimizes their consequences when they occur. Finally, SES inequalities continually impact health inequalities despite radical changes in causes of morbidity and mortality over decades (59).

According to our findings, SES disparities in health behaviors such as exercise, smoking, and drinking at baseline fail to explain the SES disparities in death due to renal diseases. Distribution of exercise (18), smoking (19), and drinking (20) are under influence of SES, while all have implications for development and progression of renal diseases (13–16). While SES has a large effect on shaping health behaviors of populations (17, 33), our study suggests that healthy behaviors at baseline are not the answer to why low SES groups are at higher risk of renal disease mortality.

While medical risk factors such as hypertension and diabetes also increased risk of renal disease mortality, they did not mediate SES disparities in renal disease mortality. Diabetes and hypertension, which are leading causes of chronic kidney disease (1), are more common among minorities and low SES individuals (23–28). Thus, at least some of the SES gradient in death due to renal disease may be due to chronic medical conditions (21–25). A well-established literature shows that diabetes is associated with chronic kidney disease (29). In fact, one of the major complications of diabetes is chronic kidney disease (30). Hypertension causes chronic kidney disease, and chronic kidney disease also increases risk of hypertension (24, 32).

Our findings that behavioral (smoking, drinking, and exercise) and medical (hypertension and diabetes) risk factors at baseline do not explain the SES disparities in death due to renal disease highlights a need for future research on other potential mechanisms of such effects. Although we did not find a mediational path, our findings have policy and clinical implications for reducing the SES gradient in death due to renal disease. Policies and programs should help people maintain health behaviors and avoid hypertension and diabetes, as they are independent risk factors for renal disease mortality.

Although other mechanisms may also be involved, SES should be considered a root cause of disparities in renal disease mortality in the United States. Factors outside baseline health behaviors and medical risk factors may contribute as mechanisms behind such effects. The health care system and health care use may be one explanation which needs future research.

Our findings suggest that SES shapes disparities in renal disease mortality and this effect is not simply because low-income people have poor health behaviors and additional medical risk factors at baseline. While considerable information exists on SES as a fundamental cause of disparities, less is known about mechanisms by which SES influences mortality across countries (60–62).

### Study Limitations

Despite the unique contribution that this paper makes to the literature, the results should be interpreted with consideration of the study limitations. The major limitation of the study was lack of any measure of kidney disease at baseline and over time. In addition, measurement bias is possible, particularly for medical risk factors. Hypertension and diabetes were measured using self-reported data. Furthermore, medical risk factors of renal disease were not comprehensive (63). Further research may use medical records to verify history of medical conditions. Future studies should also assess how changes of SES, behaviors, and CMC over time explain disparities in kidney diseases. Future research may also benefit from biological markers of kidney function. This study did not include access to health care, which may partially explain why low SES individuals and minority people have higher burden of chronic kidney disease (8). While studies have explored the role of the health care system as possible mediators of such disparities (64, 65), we did not have information on health care access or utilization in our study. Some major strengths of this study included large sample size, long-term follow-up, and recruitment of a nationally representative sample.

### Conclusions

To conclude, income disparities exist in deaths due to renal diseases over a 25-year period. Such income disparities, however, cannot be easily explained by behavioral (smoking, drinking, and exercise) and medical (diabetes and hypertension) risk factors. These findings extend the existing literature on SES disparities in renal disease in the United States. This is particularly important as the SES gradient in chronic kidney disease is a major challenge in the United States (66).

## Ethics Statement

All procedures followed were in accordance with the ethical standards of the responsible committee on human experimentation (institutional and national) with the Helsinki Declaration of 1975, as revised in 2000. Informed consent was obtained from all participants included in the study.

## Author Contributions

SA designed the study, analyzed the data, and drafted the paper. MML contributed to the paper and revised the manuscript. Both authors approved the final draft.

## Conflict of Interest Statement

The authors declare that the research was conducted in the absence of any commercial or financial relationships that could be construed as a potential conflict of interest.
